# The Clinical Benefits of Adding a Third Dimension to Assess the Left Ventricle with Echocardiography

**DOI:** 10.1155/2014/897431

**Published:** 2014-05-15

**Authors:** Luigi P. Badano

**Affiliations:** Department of Cardiac, Thoracic and Vascular Sciences, School of Medicine, University of Padua, Via Giustiniani 2, 35123 Padua, Italy

## Abstract

Three-dimensional echocardiography is a novel imaging technique based on acquisition and display of volumetric data sets in the beating heart. This permits a comprehensive evaluation of left ventricular (LV) anatomy and function from a single acquisition and expands the diagnostic possibilities of noninvasive cardiology. It provides the possibility of quantitating geometry and function of LV without preestablished assumptions regarding cardiac chamber shape and allows an echocardiographic assessment of the LV that is less operator-dependent and therefore more reproducible. Further developments and improvements for widespread routine applications include higher spatial and temporal resolution to improve image quality, faster acquisition, processing and reconstruction, and fully automated quantitative analysis. At present, three-dimensional echocardiography complements routine 2DE in clinical practice, overcoming some of its limitations and offering additional valuable information that has led to recommending its use for routine assessment of the LV of patients in whom information about LV size and function is critical for their clinical management.

## 1. Introduction

Quantitation of left ventricular (LV) size, geometry, and function represents the most frequent indication for an echocardiographic study and is pivotal for patient evaluation and management. Indication to cardiac surgery [1], to treatment initiation or suitability for device implantation in systolic heart failure [2], or to discontinuing cardiotoxic medications [3] is among the most critical decisions that rely on an accurate LV geometry and function assessment. Although conventional two-dimensional echocardiography (2DE) is by far the most used imaging modality to assess LV geometry and function in the clinical routine, its accuracy and reproducibility remain suboptimal, particularly when compared to other imaging modalities [4].

The advent of three-dimensional echocardiography (3DE) represents a real breakthrough in cardiovascular ultrasound. Advancements in computer and transducer technology permit the acquisition of 3D data sets with adequate spatial and temporal resolution for assessing most of cardiac pathologies. 3DE enables the visualization of cardiac structures from virtually any perspective, providing a more anatomically sound and intuitive display, as well as an accurate quantitative evaluation of anatomy and function of heart valves [5–10]. In addition, 3DE overcomes geometric assumptions and enables an accurate quantitative and reproducible evaluation of cardiac chambers [11, 12], thus offering solid elements for patient management [13, 14]. Furthermore, 3DE is the only imaging technique based on volumetric scanning able to show moving structures in the beating heart, in contrast to cardiac magnetic resonance (CMR) or cardiac computed tomography (CT), which are based on postacquisition 3D reconstruction from multiple tomographic images and displaying only 3D rendered snapshots.

Data regarding clinical applications of 3DE are burgeoning and gradually capturing an established place in the noninvasive clinical assessment of anatomy and function of cardiac structures. Recently, joint European Association of Echocardiography and American Society of Echocardiography recommendations have been published, aiming to provide clinicians with a systematic approach to 3D image acquisition and analysis [15]. Among the established indications for the clinical use of 3DE, the assessment of LV geometry and function is the one with more evidence.

This spotlight paper is aimed at summarizing the state-of-the-art 3DE applications to assess LV geometry and function, emphasizing the advantages of 3DE over conventional 2DE and its current limitations (Table 1).

## 2. Instrumentation

**Figure 1:**
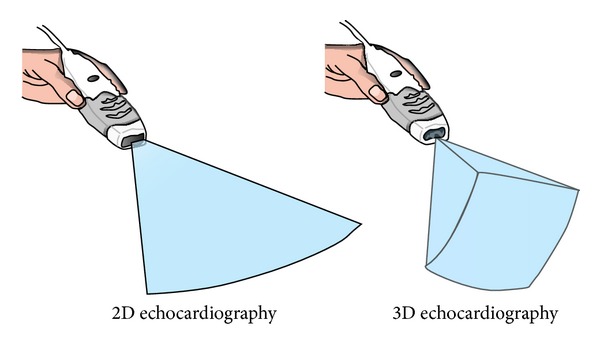
Two-dimensional echocardiography is a tomographic technique which provides “flat” views of the heart and great vessels whose thickness is fixed and related to the piezoelectric element vertical dimension. Three-dimensional echocardiography is based on real-time volumetric imaging that allows acquisition of pyramidal data sets.

The milestone in the history of 3DE has been the development of fully sampled matrix array transthoracic transducers based on advanced digital processing and improved image formation algorithms which allowed the operators to obtain on-cart transthoracic (TTE) real-time volumetric imaging with shorter acquisition time, higher spatial and temporal resolution (Figure 1) [16]. Further technological developments (i.e., advances in miniaturization of the electronics and in element interconnection technology) made it possible to insert a full matrix array into the tip of a transesophageal probe and provide transesophageal (TOE) real-time volumetric imaging [17].

### 2.1. Comparison between 2DE and 3DE Ultrasound Imaging

**Figure 2:**
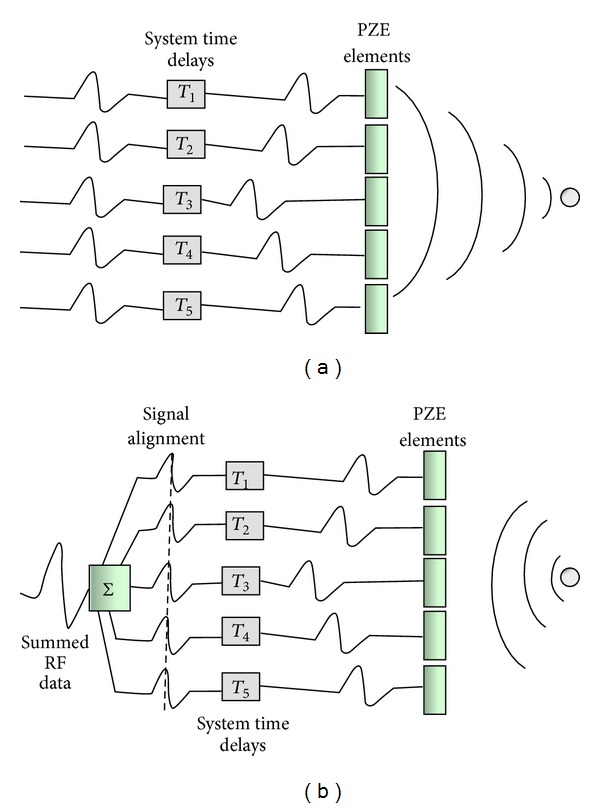
Schematic drawing of beamforming using a conventional 2D phased array transducer. During transmission (a), focused beams of ultrasound are produced by pulsing each piezoelectric element with precalculated time delays (i.e., phasing). During reception (b), focusing is achieved by applying selective delays at echo signals received by the different piezoelectric elements in order to create isophase signals that will be summed in a coherent way.

A conventional 2D phased array transducer is composed of multiple piezoelectric elements electrically isolated from each other and arranged in a single row. Individual ultrasound wave fronts are generated by firing individual elements in a specific sequence with a delay in phase with respect to the transmit initiation time. Each element adds and subtracts pulses to generate a single ultrasound wave with a specific direction that constitutes a radially propagating scan line (Figure 2). The linear array can be steered in two dimensions—vertical (axial) and lateral (azimuthal)—while the resolution in the *z* axis (elevation) is fixed by the thickness of the tomographic slice, which, in turn, is related to the vertical dimension of piezoelectric elements.

**Figure 3:**
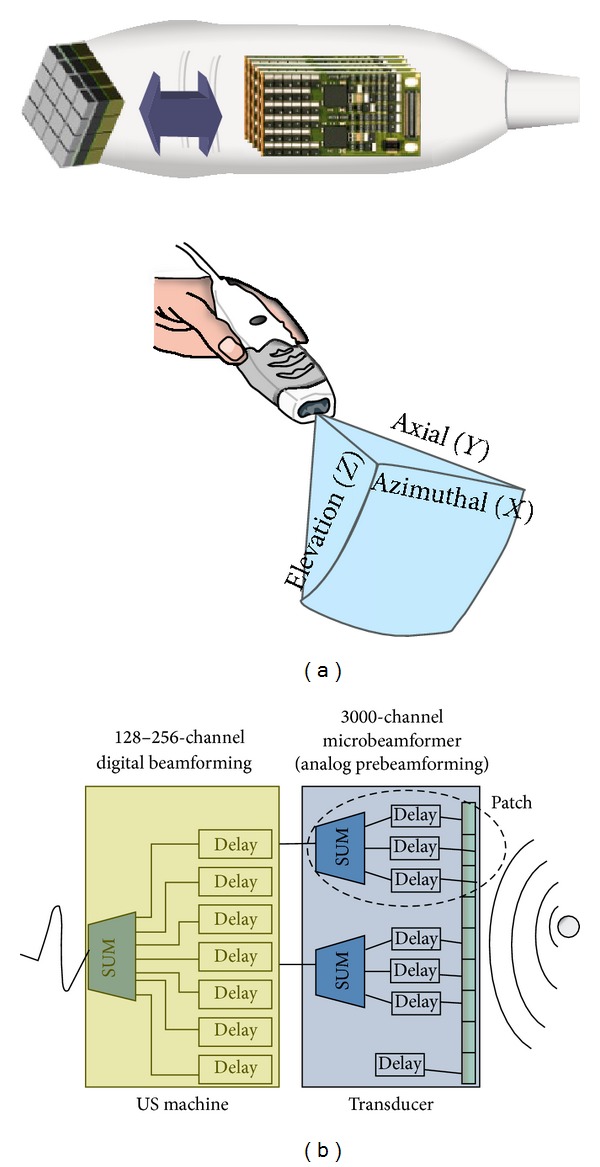
(a) Schematic drawing of a full matrix array transducer where about 3000 acoustically independent piezoelectric elements are arranged in row and columns and used to steer the beam electronically. This matrix arrangement of piezoelectric elements allows their phasic firing to produce an ultrasound beam that can be steered in vertical (axial), lateral (azimuthal), and anteroposterior (elevation) directions in order to acquire a volumetric (pyramidal) data set. (b) Beamforming with 3D matrix array transducers. To save power and electronic circuitry needs (costs) and reduce the connection cable size the beamforming and steering processes have been split into two: the transducer and the ultrasound machine levels. The transducer contains the piezoelectric elements arranged in a matrix array, interconnection technology and integrated analog circuits (DELAY) to control transmit and receive signals using different subsections of the matrix (patches) to control analog prebeamforming and fine steering. Signals from each patch are summed in order to reduce the number of digital lines in the coaxial cable that connects the transducer to the ultrasound system from 3000 to the conventional size of 128–256 channels. At the ultrasound machine level, analog-to-digital (A/D) convertors amplify, filter, and digitize the elements signals. The resulting digital signals are focused (coarse steering) using digital delay (DELAY) circuitry and summed together (*Ξ*) to form the received signal from a desired object.

Currently, 3DE matrix array transducers are composed of about 3000 independent piezoelectric elements with operating frequencies ranging from 2 to 4 MHz and 5 to 7 MHz for TTE and TOE, respectively. These piezoelectric elements are arranged in a matrix configuration within the transducer (Figure 3(a)) in order to steer the ultrasound beam electronically. The electronically controlled phasic firing of the elements in that matrix generates a scan line that propagates radially and can be steered both laterally (azimuth) and in elevation in order to acquire a volumetric pyramid of data. The main technological breakthrough which allowed manufacturers to develop the matrix transducers has been the miniaturization of electronics that allowed the development of individual electrical interconnections for every piezoelectric require element which could be independently controlled, both in transmission and in reception. On the other hand, the microbeamforming allows the same size of the 2D cable to be used with 3D probes, despite the large number of digital channels required for these fully sampled elements to be connected.

Beamforming is a technique used to process signals in order to produce directionally or spatially selected signal sent or received from arrays of sensors. In 2DE, all the electronic components for the beamforming (high-voltage transmitters, low-noise receivers, analog-to-digital converter, digital controllers, and digital delay lines) are in the system and consume a lot of power (around 100 W and 1,500 cm^2^ of personal computer electronics board area). If the same beamforming approach would have been used for matrix array transducers used in 3DE, it would require around 4 kW power consumption and a huge PC board area to accommodate all the needed electronics. To reduce both the power consumption and the size of the connecting cable, several miniaturized circuit boards are incorporated into the transducer, allowing partial beamforming to be performed in the probe (Figure 3). The 3000 channel circuit boards within the transducer control the fine steering by delaying and summing signals within subsections of the matrix, known as patches. This allows reducing the number of the digital channels to be put into the cable that connects the probe to the ultrasound system from 3000 to the conventional 125–256. Coarse steering is controlled by the ultrasound system where the analog-to-digital conversion occurs using digital delay lines (Figure 3).

Additionally, developments in transducer technology have resulted in a reduced transducer footprint, improved side-lobe suppression, increased sensitivity and penetration, and implementation of harmonic capabilities that can be used for both gray-scale and contrast imaging. The most recent generation of matrix transducers are significantly smaller than the previous ones and the quality of 2D and 3D imaging has improved significantly, allowing a single transducer to acquire both 2D and 3DE studies, as well as acquiring the whole left ventricular cavity in a single beat.

## 3. Image Acquisition and Display 

Currently, 3D data set acquisition can be easily implemented into standard echocardiographic examination either by switching among 2D and 3D probes or, with newest all-in-one-probes, by switching between 2D and 3D modalities available in the same probe. The latter probes are also capable of providing single-beat full-volume acquisition, as well as real-time 3D color Doppler imaging.

**Figure 4:**
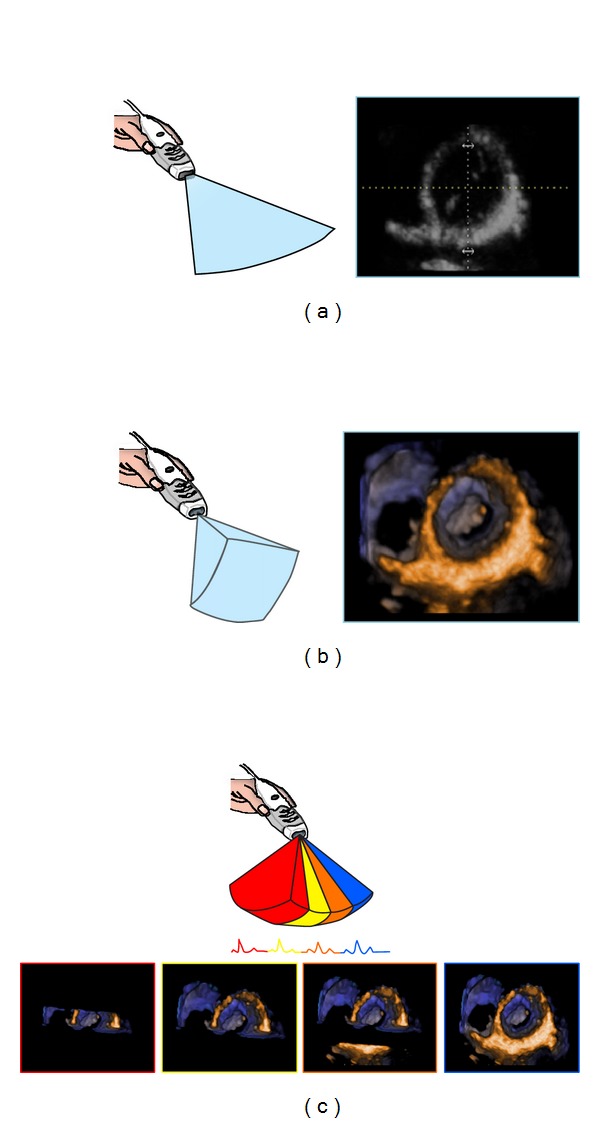
Schematic representation of two-dimensional (i.e., tomographic (a)) and single-beat three-dimensional (i.e., volumetric (b)) of the left ventricular short axis at mitral valve level. Volumetric rendering displays many more details and allows better appreciation of spatial relationship among cardiac structures. (c) shows the multibeat acquisition modality: two to six consecutive single-beat subvolumes are stitched together to obtain a full-volume with higher spatial and temporal resolution.

At present two different methods for 3D data acquisition are available: “real-time” (or “live” 3D mode) and multibeat 3D mode (Figure 4) [18]. In the real-time mode, a thin sector of a pyramidal 3D volume data set is obtained and visualized live, beat after beat as during 2D scanning. Imaging is usually available in several fashions, as narrow volume, zoom, wide-angle (full-volume), and color-Doppler modalities. Heart dynamics is shown in a realistic way, with instantaneous online volume rendered reconstruction. It allows fast acquisition of dynamic pyramidal data structures from a single acoustic view that can encompass the entire heart without the need of reference system and electrocardiographic (ECG) and respiratory gating. Real-time imaging is time-saving for both data acquisition and analysis. Although this acquisition mode overcomes rhythm disturbances or respiratory motion limitations, it still suffers from relatively poor temporal and spatial resolutions.

Conversely, multibeat acquisition is realized through sequential acquisitions of narrow smaller volumes obtained from several ECG-gated consecutive heart cycles (from 2 to 6) that are subsequently stitched together to create a single volumetric data set. It provides large data sets with high temporal and spatial resolution, but more prone to artifacts due to patient or respiratory motion or irregular cardiac rhythms. The most appropriate acquisition mode for the specific clinical setting will be chosen in each individual case.

**Figure 5:**
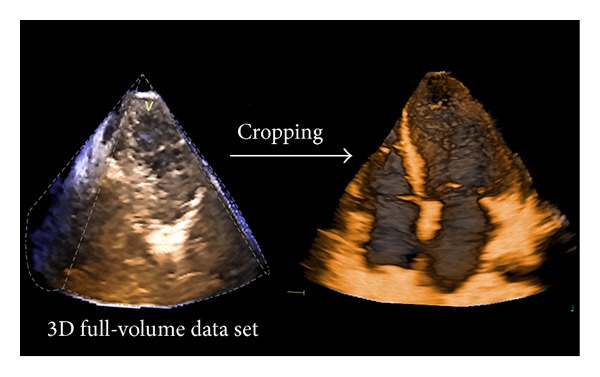
Cropping allows obtaining stereoscopic images of the cardiac structures contained in a pyramidal 3D data set.

**Figure 6:**
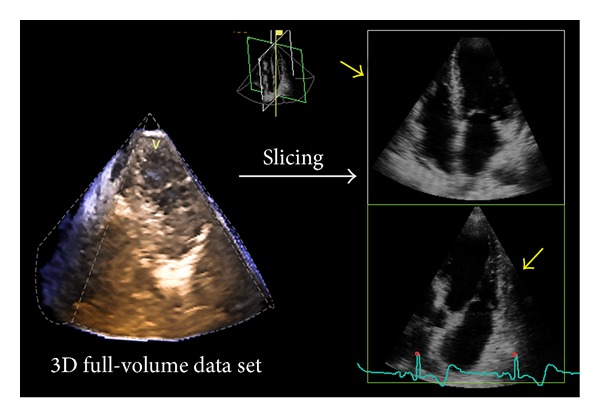
The 3D data set can be sliced in several 2D cut planes to obtain multiple views of that particular chamber (in the figure a 4–chamber view, upper panel, and an orthogonal view of the left ventricle and the left atrium, lower panel). Simultaneous visualization of orthogonal 2D slices enables the assessment and comparison of wall motion at every segment level of the ventricle, precious for assessing severity and extension of hypertrophy or regional wall motion evaluation both at rest and during stress.

3D data sets can be sectioned in several planes and rotated in order to visualize the cardiac structure of interest from any desired perspective, irrespective of its orientation and position within the heart. This allows the operator to easily obtain unique visualizations that may be difficult or impossible to achieve using conventional 2DE (e.g.,* en face* views of the tricuspid valve or cardiac defects). Two main actions are undertaken by the operator to obtain the desired view from a 3D volumetric data set: cropping and slicing. Similar to what the anatomists or the surgeons do to expose an anatomic structure within a 3DE data set, the operator should remove the surrounding chamber walls. This process of virtually removing the irrelevant neighbouring tissue is called cropping (Figure 5) and can be performed either during or after acquisition. In contrast with 2D images, displaying a cropped image requires also the definition of the viewing perspective (i.e., since the same 3D structure can be visualized* en face* from either above or below, as well as from any desired view angle) [1]. Slicing refers to a virtual “cutting” of the 3D data set into one or more (up to twelve) 2D (tomographic) grey-scale images (Figure 6).

**Figure 7:**
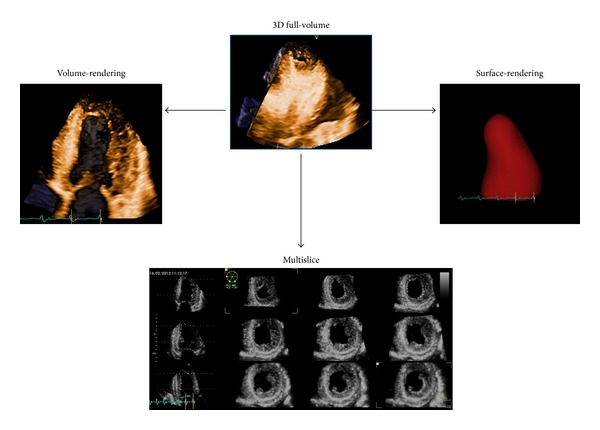
From the same pyramidal three-dimensional data set, the left ventricle can be visualized using different display modalities: volume rendering, for visualizing morphology and spatial relationships among adjacent structures; surface rendering, for quantitative purposes; and multislice (multiple two-dimensional tomographic views extracted automatically from a single 3D data set) for morphological and functional analysis at different regional levels.

**Figure 8:**
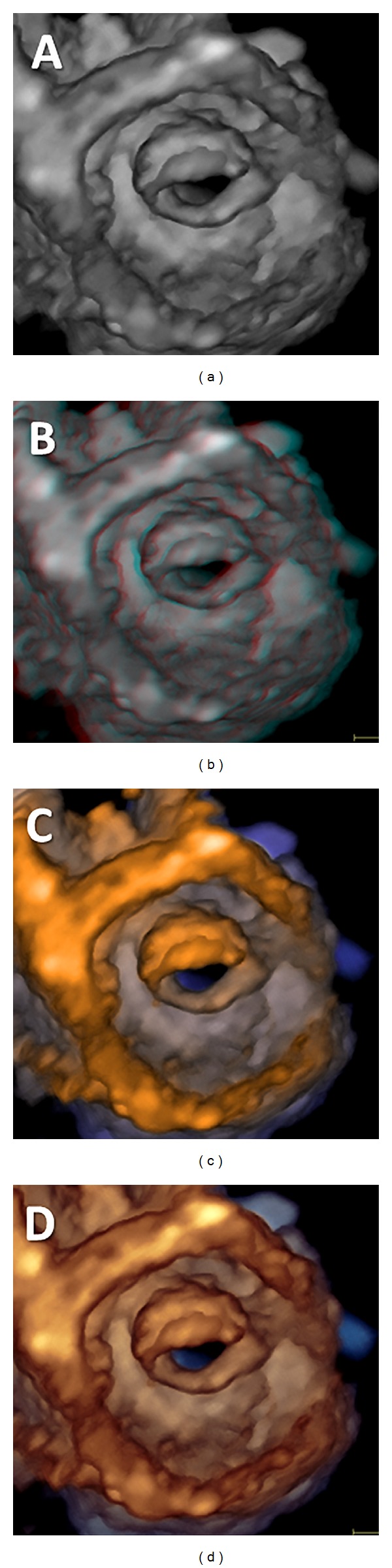
Examples of volume rendering in mitral stenosis using various depth-encoding colorizations: (a) grey; (c) bronze-blue; (d) copper-blue. (b) illustrates the stereo rendering.

**Figure 9:**
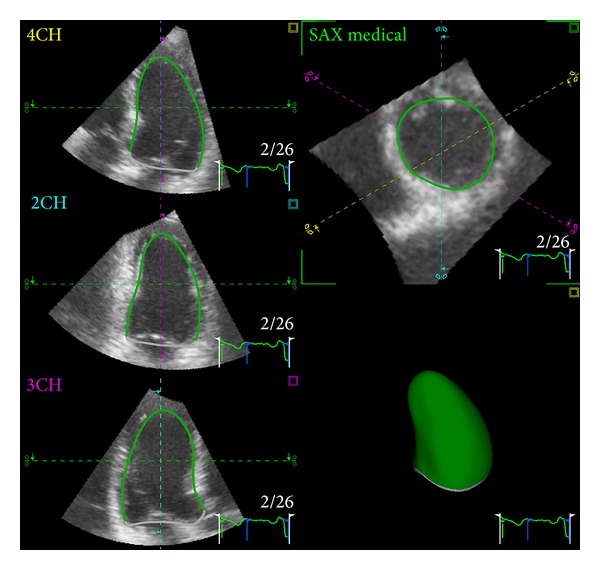
Surface rendering display of left ventricular volume. After having identified the endocardial border on the 3 apical slices, the software automatically detects the whole endocardial surface. The accuracy of endocardial border detection on the whole left ventricular circumference can be checked on the transverse slice which can be moved up and down along the left ventricular long axis. Once confirmed by the operator, a 3D cast of the left ventricle is developed and the volume is measured by counting the voxels within the volume.

Acquisition of volumetric images generates the technical problem of rendering the depth perception on a flat, 2D monitor. 3D images can be visualized using three display modalities: volume rendering, surface rendering, and tomographic slices (Figure 7). In volume rendering modality, various color maps are applied to convey the depth perception to the observer. Generally, lighter shades (e.g., bronze, Figure 8) are used for structures closer to the observer, while darker shades (e.g., blue, Figure 8) are used for deeper structures. Surface rendering modality displays the 3D surface of cardiac structures, identified either by manual tracing or by using automated border detection algorithms on multiple 2D cross-sectional images of the structure/cavity of interest (Figure 9). This stereoscopic approach is useful for the assessment of shape and for a better appreciation of geometry and dynamic function during the cardiac cycle. Finally, the pyramidal data set can be automatically sliced in several tomographic views simultaneously displayed (Figure 6). Cut planes can be orthogonal, parallel, or free (any given plane orientation), selected as desired by the echocardiographer for obtaining optimized cross-sections of the heart in order to answer specific clinical questions and to perform accurate and reproducible measurements.

In the following sections, we will review the current clinical applications of 3DE to assess the geometry and function of the left ventricle and discuss advantages of the technique over conventional 2DE, as well as its limitations.

## 4. Assessment of Left Ventricular Geometry and Function with 3DE

### 4.1. Quantification of LV Volumes and Ejection Fraction

To quantitate LV volumes and ejection fraction with 2DE, biplane imaging (i.e., acquisition of both 4- and 2-chamber apical views of the LV) is recommended [19]. However, this approach has several limitations. In many patients, it is difficult to acquire high-quality images in both of these views and to ensure that the imaging planes are truly perpendicular to each other. Moreover, the endocardium should be manually traced in both views and, therefore, LV volume measurement by 2DE is highly dependent on user's experience. Finally, 2DE uses only partial information contained in 2 predefined cross-sections of the LV to assess global myocardial function and relies on geometrical assumptions about the shape of the LV that may not be necessarily valid in all patients.

**Figure 10:**
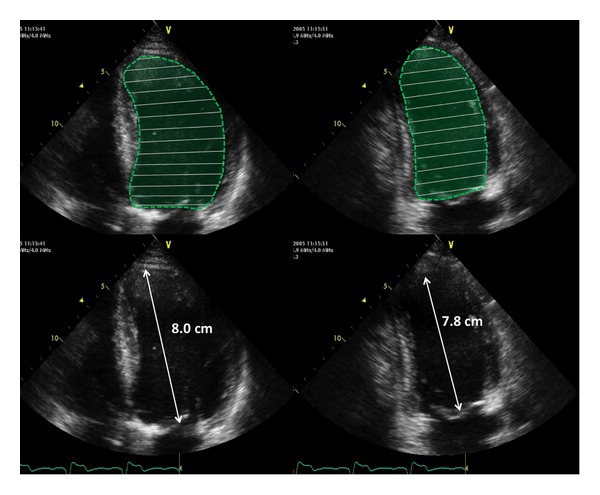
Schematic representation of the biplane disc summation algorithm based on the measurement of left ventricular areas and long axis lengths on 4- and 2-chamber two-dimensional views of the left ventricle. To obtain accurate calculation of left ventricular volume the difference between the long axes measured in 4- and 2-chamber apical views should not be larger than 10%.

The greatest advantage of 3DE in the evaluation of the LV is that, with this technique, the commonest causes of LV volume underestimation with conventional 2DE (i.e., foreshortening of the LV long axis, plane position errors, and geometrical assumptions about LV shape) are no longer real issues (Figure 9) [20, 21]. With 3DE, only one acquisition of the LV is required to obtain volumes and ejection fraction (Figure 10). The acquisition is usually performed from the apical approach and requires that the whole LV is included within the 3D data set. LV data set analysis can be performed using computerized automated or semiautomated endocardial surface detection software, which do not rely on any geometric assumption about LV geometry and require only minimal human intervention, therefore improving measurement reproducibility.

3D TTE has been extensively validated against CMR (Table 2) and has been demonstrated to be more time-saving, reproducible, and accurate than conventional 2DE for LV volumes and ejection fraction measurement. In most publications, 3D TTE has been shown to slightly underestimate both LV end-diastolic and end-systolic volumes in comparison with those measured with CMR (Tables 2 and 3). A recent meta-analysis of 23 studies comparing 3D TTE with CMR volumes and ejection fraction demonstrated biases of −19 ± 34 mL, −10 ± 30 mL, and −1 ± 12% for LV end-diastolic and end-systolic volumes, and ejection fraction, respectively [22]. Among the few studies that did not exclude patients for poor image quality, the negative volume biases were slightly larger: −29 ± 38 mL, −18 ± 34 mL, and +3 ± 16% for end-diastolic and end-systolic volumes, and ejection fraction, respectively. As compared to 2D TTE, 3D TTE performed favorably in terms of accuracy and reproducibility. Among the 9 studies including both echocardiographic modalities, negative volume biases were larger for 2D TTE than for 3D TTE: for end-diastolic volume, −48 ± 56 mL versus −16 ± 31 mL; for end-systolic volume, −28 ± 46 mL versus −10 ± 26 mL; and for ejection fraction, 1 ± 14% versus 0 ± 9%.

In another recent study, 36 prospectively enrolled patients underwent 3D TTE, 2D TTE, CMR, CT with contrast, and invasive cine ventriculography [23]. Both 3D TTE and 2D TTE significantly underestimated LV volumes as compared to CMR: end-diastolic volumes were lower by 18 and 26 cc, respectively, while CT slightly overestimated end-diastolic volume by 6 cc. For ejection fraction, both 3D TTE and 2D TTE values correlated equally well with CMR values (*r* = 0.79 for both), with CT outperforming both (*r* = 0.89). 3D TTE LV ejection fraction values were also less reproducible than those derived from CT. One might speculate that TTE's apparent disadvantage in this study is related to its operator dependence, during both acquisition and image analysis, given that the CT software automatically determined volumes while the specific 3D TTE software used in this study (4D LV function, TomTec Imaging Systems, Unterschleißheim, D) requires tracings of the endocardial borders at both end-diastole and end-systole.

The systematic underestimation of LV volumes by 3D TTE stems in part from its lower spatial resolution as compared to CMR. By convention, when CMR volumes are traced according to Simpson's method of disks, the trabeculae are included in the LV cavity. With 3D TTE, it is often difficult to clearly identify the endocardial-trabecular border, and as a result, one may instead trace the blood-trabecular interface. In a systematic review of sources of error, it was shown that agreement between 3D TTE and CMR measurements improved when the trabeculae were excluded from the LV cavity [24]. In this study, agreement between 3D TTE and CMR also improved with increasing investigator experience; experienced observers tended to place their 3D TTE borders as far outward as possible.

**Figure 11:**
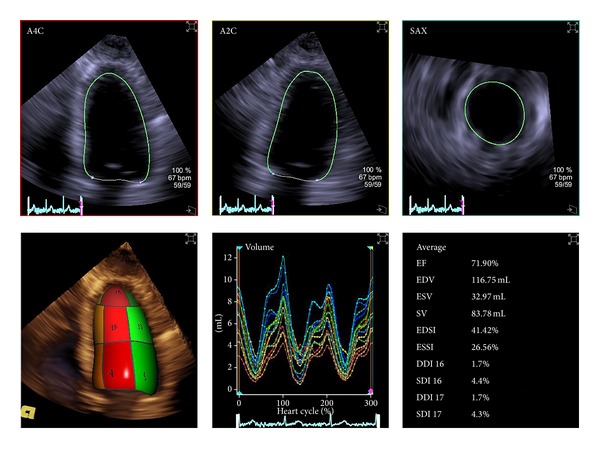
Single-beat full-volume acquisition and automatic endocardial border detection allow fast assessment of left ventricular volume and ejection fraction with the possibility of averaging results over multiple cardiac beats.

A recent meta-analysis of studies evaluating 3D TTE for LV volumes demonstrated that 3D TTE was superior to older techniques requiring transducer rotation and reconstruction [22]. Although single-beat acquisition for quantification of LV volumes is feasible [25, 26], its low temporal resolution results in underestimation of EF as compared to 2- and 4-beat acquisition [11]. This is due to the misidentification of the end-systolic frame at low frame rates. The method of data analysis also affects accuracy. Most current software programs perform semiautomated quantification of volumes, requiring the user to identify anatomical landmarks (usually including the apex and mitral annulus) on one or more longitudinal slices of the LV derived from the 3D dataset. The user is then able to edit the endocardial borders displayed by the program. Several studies have shown that when endocardial borders are traced and/or adjusted on more than 2 cut planes, accuracy of volume quantification is improved [27–29]. This is likely due to the fact that fewer geometric assumptions are required. With programs that perform automated border detection, software sensitivity settings also influence volumes. Lower sensitivity causes the border to be drawn closer to the endocardial-trabecular border, rather than at the blood-trabecular interface, increasing volumes and resulting in less bias as compared to CMR [30]. Despite all the advantages of 3D TTE, its use remains limited mostly to academic centers. Acquisition and processing of 3D datasets require specialized skills, which sonographers and physicians may not obtain during their formal training. Moreover, quantification of LV volumes and ejection fraction with currently available 3D software is time consuming. Fortunately, software programs that perform fully automated LV endocardial detection are being developed and appear promising [31]; their ease of use will likely expand the role of 3D TTE in everyday practice (Figure 11).

3D TTE is a robust technique for quantifying LV volumes and ejection fraction in patients with abnormal LV geometry. Regional wall motion abnormalities, including those due to myocardial infarction, do not compromise the accuracy of LV volume and ejection fraction measurement [32–35]. 3D TTE volumes are generally accurate even in very dilated and aneurysmal ventricles [36–38], provided that the sonographer takes care to include the entire ventricle in the pyramidal data set.

In a recent meta-analysis Dorosz et al. [39] found significantly larger biases and limits of agreement for 2D TTE (−48 ± 56 mL, −28 ± 46 mL, and 0.1 ± 14% for LV volumes and ejection fraction, resp.) than for 3D TTE (−19 ± 34 ML, −10 ± 30 mL, and 0.6 ± 12%) when both were compared with CMR. These data, together with the superior reproducibility of 3D TTE, should warrant its use as the preferred echo technique to assess LV size and function.

For years, the usefulness of 3D TTE in everyday practice was limited by the absence of reference values for LV chamber volumes and ejection fraction. Recently, several publications [40–44] have addressed this gap in the literature (Table 4). Some of the variation in the reference ranges from study to study is likely attributable to differences in echocardiographic equipment and analysis software, as well as heterogeneity in measurement techniques [45]. Despite the fact that the LV volumes obtained in normal subjects are significantly larger with 3DE than with 2DE, the LV ejection fraction is similar [41]. The largest of these studies [44] explicitly stratified subjects according to ethnicity, finding that LV volumes were smaller among Asian Indians than White Europeans, while ejection fraction did not differ. The EchoNORMAL (the echocardiographic normal ranges meta-analysis of the left heart) collaboration study carried out by the University of Aukland (New Zealand) is a meta-analysis of echo measurements obtained from 23301 normal subjects collected from several echo labs around the world and will provide ethnicity specific reference values for most of the conventional and 3D echocardiographic measurements [46]. In most of the studies that measured LV geometry and function in normal subjects [40, 41, 43, 44], weak to moderate negative correlations were seen between age and LV volumes, while LV ejection fraction showed either no change or a significant increase with age [41]. These findings are in keeping with those of prior CMR studies [47, 48]. The ongoing normal reference ranges for echocardiography (NORRE) study has been designed to establish normal values for a variety of 2D and 3D TTE parameters in a population of 1100 Caucasian Europeans, ranging in age from 25 to 75 [49].

### 4.2. Assessment of Regional Left Ventricular Function

**Figure 12:**
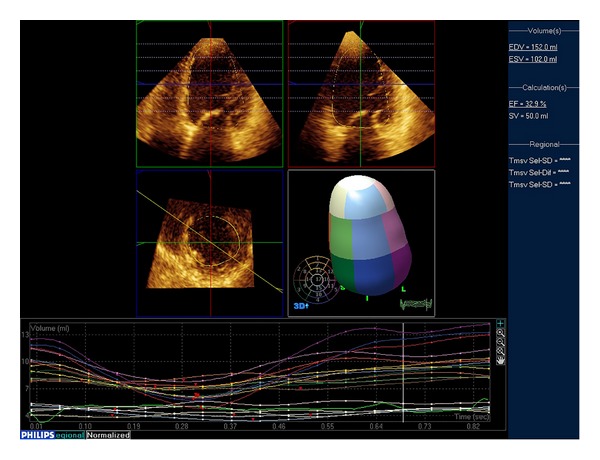
The full-volume of the left ventricle can be divided into 16 or 17 regional pyramidal subvolumes whose base is on the endocardium (different colours identify different segments) and the apex is in the center of gravity within the cavity of the left ventricle. The volume changes of individual regional pyramidal subvolume can be tracked throughout the cardiac cycle (see the time-volume curves in the lower part of the figure) and a “regional ejection fraction” can be measured.

Unlike 2D TTE, which requires the sonographer to move and rotate the probe in order to acquire images of all LV walls from the parasternal and apical windows, 3D TTE can image all walls in a single volume acquisition. Moreover, foreshortening is not a problem anymore with 3D TTE, and 2D slices obtained from the postprocessing of the 3D data set can be rotated and reoriented at anytime after acquisition. In addition, a number of 2D views (slices) can be simultaneously displayed from the acquired 3D data set, providing a comprehensive visualization of LV endocardial motion and myocardial thickening (Figure 7). For these reasons, 3D TTE is particularly well-suited to assess the location and extent of regional wall motion abnormalities of the LV. Regional LV volumes by 3D TTE (Figure 12) have been shown to correlate well with CMR (*r* values generally 0.8 and higher) [50]. Thorstensen et al. [51] reported in patients with recent myocardial infarction a reasonable correlation (*r* = 0.74) between wall motion score index by 3D TTE and the extent of delayed gadolinium enhancement by CMR. Among patients with suboptimal 3DTTE image quality, administration of echocardiographic contrast has been shown to improve agreement of wall motion assessment with CMR [52].

In the area of stress echocardiography, 3D TTE is advantageous because it requires only a single apical acquisition at peak stress, whereas 2D TTE requires at least 3 (apical 2-, 3-, and 4-chamber views). This would be particularly useful in patients with rapid heart rate recovery after exercise. Furthermore, during offline postprocessing, it is possible to view images in a multitude of planes and reorient them to determine the extent of wall motion abnormalities and to rule out artifacts. A number of studies have demonstrated that exercise and pharmacologic stress testing with 3D TTE are feasible, with more rapid image acquisition and higher interobserver agreement in wall motion interpretation as compared to 2D TTE [53–58]. The sensitivity of 3D TTE to significant coronary artery disease, based on an invasive angiography reference standard, has been found equal to or better than that of 2D TTE in most [53, 55, 57] but not in all studies [59]. Inadequate segment visualization is most common in the anterior and lateral walls [58], but administration of an ultrasound contrast agent may significantly improve image quality [60]. In order to optimize detection of wall motion abnormalities, it is important to maximize temporal resolution (i.e., use a volume rate as high as possible) and to ensure adequate breath holds to avoid artifacts. Specialized software that helps the echocardiographer identify corresponding planes at rest and stress, so that each wall may be evaluated against itself, has been shown to improve interobserver agreement for wall motion analysis [61].

### 4.3. Quantification of Left Ventricular Mass

**Figure 13:**
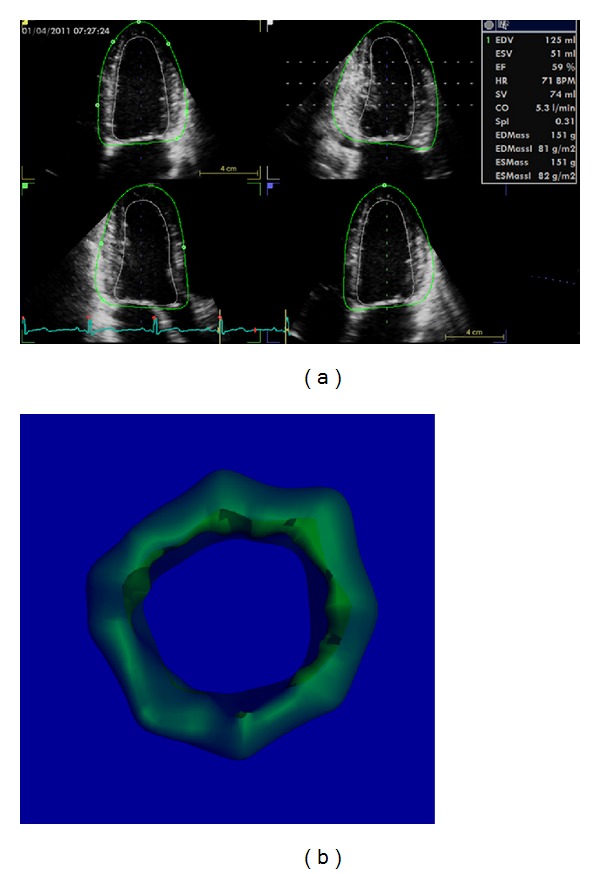
Left ventricular mass measurement using three-dimensional echocardiography. Using automated or semiautomated endocardial and epicardial boundary detection endocardial and epicardial volumes are measured (a). By subtracting the left ventricular cavity volume from the epicardial volume, the volume of myocardium is obtained (b). Left ventricular mass is calculated by multiplying myocardial volume by its specific gravity (1.05 g/cm^3^).

For quantification of LV mass, both the endocardial and epicardial surfaces must be outlined to measure LV myocardial volume (Figure 13). By convention, as with CMR tracings, the papillary muscles are part of the LV cavity (i.e., not included in the myocardial volume). LV myocardial volume is then multiplied by the density of cardiac muscle (1.05 g/mL) to calculate LV mass. With 3D TTE datasets, LV mass analysis is typically done offline using semiautomated software (Figure 13).

Several studies have compared 3D TTE to 2D TTE and CMR and have generally found that LV mass measured by 3D TTE correlates better with CMR results [62–72]. Unlike 2D TTE, 3D TTE does not systematically underestimate mass; likely because foreshortening of the LV long-axis dimension is generally avoided [63]. Furthermore, 3D TTE has better interobserver agreement than 2D TTE [63, 64, 69]. Even in the presence of wall motion abnormalities, abnormal LV geometry secondary to congenital heart disease or hypertrophic cardiomyopathy, 3D TTE-based measurement of LV mass is relatively accurate as compared to CMR [32, 66, 70] although a recent meta-analysis has suggested that LV mass measurements are more likely to be underestimated in patients with cardiac disease than in healthy volunteers [67].

Interestingly, M-mode echocardiography, a method commonly used to diagnose LV hypertrophy in clinical practice, has been shown in some studies to overestimate LV mass in comparison to 3D TTE [41, 72–74]. This is likely because M-mode relies heavily on geometric assumptions and is greatly influenced by the acquisition plane. M-mode wall thickness measurements are usually obtained near the base of the heart at the level of the papillary muscles, where the LV wall tends to be thickest, whereas the actual wall thickness decreases gradually from base to apex. In a small study of LV mass in patients with abnormal LV geometry due to congenital heart disease, M-mode correlated poorly with CMR (*r* = 0.38), while 3D TTE performed well (*r* = 0.94) [75].

Two studies have reported reference values for 3D LV mass and LV mass/end-diastolic volume ratio in Japanese and Italian cohorts (Table 3) [40, 41].

### 4.4. Assessment of LV Dyssynchrony

The availability of cardiac resynchronization therapy (CRT) for refractory heart failure has generated considerable interest in LV intraventricular dyssynchrony assessment. For quantitative dyssynchrony evaluation, the LV is most commonly divided into 16 or 17 segments as per the American Heart Association standard model [76], and the time to minimum systolic volume is determined for each LV segment. The standard deviation of the regional times to minimum systolic volume has been proposed as the dyssynchrony index [77]. Several small studies have suggested that an increased dyssynchrony index prior to implant is predictive of favorable response to CRT [78–81] and that placement of the LV lead near the most delayed segment may be associated with favorable LV remodeling [82], but these findings have not been consistent [83, 84]. It is clear that greater degrees of LV dysfunction are associated with higher dyssynchrony indices [77, 85–87], but this may be due to noisy, low-amplitude ejection curves as much as true dyssynchrony [21, 85]. Moreover, inter- and intraobserver reproducibility of the 3D dyssynchrony index is hindered by suboptimal image quality [78] and is less robust than that of tissue Doppler imaging [87, 88]. The low temporal resolution of 3D TTE as compared to tissue Doppler imaging is another important consideration in this context [89]. However, the lower temporal resolution does not seem to be a major drawback of second-generation 3DE scanners for LV dyssynchrony assessment [30]. Since 3DE measures regional volume changes and not regional velocities (as tissue Doppler imaging, which requires a high frame rate), it seems likely that echo systems using a temporal resolution of 20 to 30 volumes/s are adequate to sample the usual frequency of regional volume curves which is less than 10 Hz [90].

Finally, a reliable, clinically meaningful cutoff value for the 3D dyssynchrony index has yet to be established [89]. Larger, multicenter studies are needed to establish the role of 3D TTE dyssynchrony analysis in the evaluation of patients prior to CRT.

### 4.5. Novel (Research) Applications of 3D TTE for Advanced Assessment of the Left Ventricle

**Figure 14:**
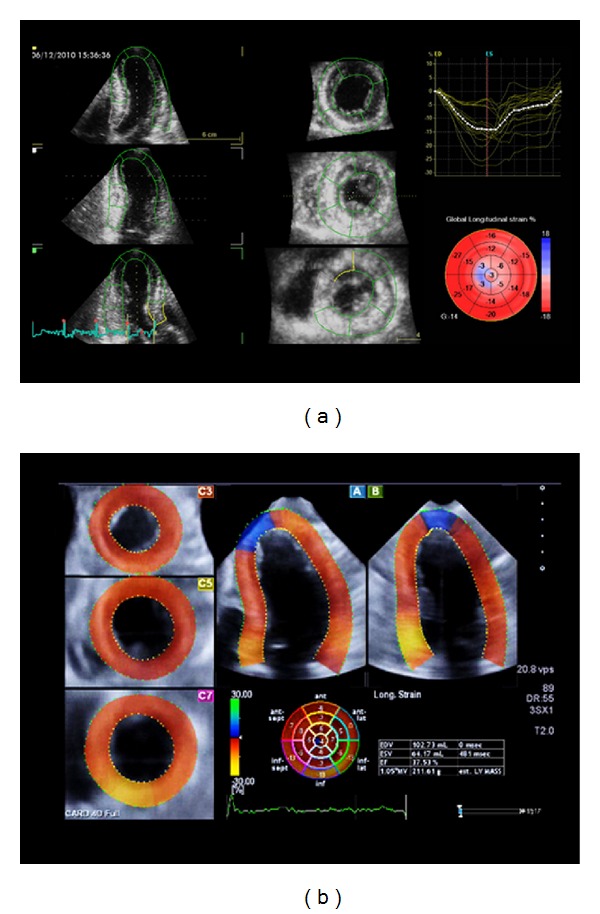
Three-dimensional speckle-tracking analysis of left ventricular longitudinal myocardial deformation using two different platforms. Results can be displayed as bull's eye plots and/or time-strain curves.

3D strain is a novel technology aimed at assessing LV myocardial deformation by analyzing the motion of myocardial speckles within the 3D LV data sets. This technology allows following the motion of speckles in the space without any assumption about the direction of their motion and the measurement of all LV deformation components (longitudinal, radial, and circumferential) plus the computation of the area strain (a composite one which includes the longitudinal and circumferential deformation) from a single apical LV 3D data set. Theoretically, 3D speckle-tracking technology should overcome the main limitations of 2D speckle tracking: the “out-of-plane” motion of speckles due to the rotation and shortening motion of the left ventricle and the need to interpolate the whole LV myocardium from the partial information contained in a limited number of thin, tomographic slices of the LV (Figure 14).

3D TTE strain acquired using speckle tracking has been studied in several clinical contexts. In patients surviving an acute myocardial infarction, higher baseline global longitudinal strain by 3D TTE is independently predictive of improvement in LV ejection fraction at 6-month follow-up [91]. Impaired circumferential, radial, and longitudinal strains have been associated with LV dilation in the setting of ischemic cardiomyopathy with reduced LV ejection fraction [92]. In a population of diabetic patients with normal LV ejection fraction, elevated hemoglobin A1c correlated with reduced global longitudinal, circumferential, and area strain [93]. A study of heart transplant recipients with preserved LV ejection fraction showed that 3D speckle-tracking global longitudinal and circumferential strain, but not 2D strain, were predictive of New York Heart Association functional class [94]. These findings suggest that 3D speckle-tracking strain could be useful in predicting outcomes and detecting subclinical disease in a variety of cardiac and systemic conditions.

To date, the main issues that are currently limiting the applicability of 3D strain in the clinical arena are (i) lack of reference values for the regional and global strain parameters for the various deformation components; (ii) lack of multicenter outcome studies assessing its additive prognostic value over the well-established 2D strain; and (iii) significant intervendor differences of 3D strain algorithms and values among vendors, preventing its routine applicability for clinical purposes [95].

Another application of 3DE that may open a complete new way to assess prognosis in patients with myocardial and valvular heart diseases is the quantitative analysis of LV shape. 3D TTE is well-suited to portray global and regional shape of the LV [96]. A 3D TTE study by Salgo et al. [97] demonstrated that, in patients with dilated cardiomyopathy, the apical and septal curvatures were diminished as compared to controls, reflecting the ventricle's overall globular shape. Mannaerts et al. [98] have described a 3D sphericity index that, in a postmyocardial infarction population, is highly predictive of adverse remodeling (progressive LV dilation). In a 3D TTE study of patients with severe mitral regurgitation, sphericity was increased as compared to controls, even in the presence of only mild LV dilation and normal ejection fraction; following successful mitral valve repair, sphericity decreased [99].

## 5. Conclusions

3DE is a novel imaging technique based on acquisition and display of volumetric data sets in the beating heart. This permits a comprehensive evaluation of LV anatomy and function from a single acquisition and expands the diagnostic possibilities of noninvasive cardiology. It provides the possibility of quantitating geometry and function of LV without preestablished assumptions regarding cardiac chamber shape and allows an echocardiographic assessment of the LV that is less operator-dependent and therefore more reproducible.

Further developments and improvements for widespread routine applications include higher spatial and temporal resolution to improve image quality, faster acquisition, processing and reconstruction, and fully automated quantitative analysis. At present, 3DE complements routine 2DE in clinical practice, overcoming some of its limitations and offering additional valuable information that has led to recommending its use for routine assessment of the LV of patients in whom information about LV size and function is critical for their clinical management.

## Figures and Tables

**Table 1:** Left ventricular assessment by 3DE.

Advantages	Limitations
(i) From a 3D data set, 2D planes can be easily realigned after the acquisition to identify LV maximum longitudinal axis avoiding apical foreshortening and optimizing volumetric quantification	(i) Accurate semiautomated or fully automated LV quantitation can only be performed on good image quality data sets usually obtained in 80–85% of routine patients
(ii) 3DE measurements of left ventricular volumes are independent of geometric assumptions about its shape	(ii) To avoid stitching artifacts, regular cardiac rhythm and patient cooperation for breath holding are essential (i.e., 3DE cannot be used in patients with irregular atrial fibrillation or frequent ectopic beats, and severely dyspnoeic or clinically unstable patients)
(iii) From a single 3D full-volume data set, the operator can quickly obtain a comprehensive assessment of LV geometry and function (volumes, sphericity, ejection fraction, regional wall motion, dyssynchrony, strain in 4 dimensions, and mass)	(iii) The intervendor consistency of 3D quantitative parameters remains an unresolved issue
(iv) When compared to cardiac magnetic resonance, 3DE is more accurate and reproducible than 2DE in assessing LV geometry (volumes, mass, and shape) and function	(iv) The relatively low temporal resolution of 3DE limits the assessment of regional wall motion during exercise and dobutamine stress echo
(v) From a 3DE data set of the left ventricle, both qualitative and quantitative assessment of regional wall motion can be obtained in a faster, more accurate, and comprehensive manner in comparison with 2DE	

**Table 2:** Differences between left ventricular volumes and ejection fraction assessed by three-dimensional echocardiography and conventional two-dimensional echocardiography in comparison with cardiac magnetic resonance.

Author	Parameter	Mean difference ± SD from CMR	
		3DE	2DE
Hof et al. [100]	End-diastolic volume (mL)	−4 ± 9	−54 ± 33
	End-systolic volume (mL)	−3 ± 18	−28 ± 28
	Ejection fraction (%)	0 ± 7	−1 ± 13

Kühl et al. [101]	End-diastolic volume (mL)	−13.6 ± 18.9	—
	End-systolic volume (mL)	−12.8 ± 20.5	—
	Ejection fraction (%)	0.9 ± 4.4	—

Hudsmith et al. [102]	End-diastolic volume (mL)	−4.1 ± 29	−23 ± 86
	End-systolic volume (mL)	−3.5 ± 33	−19 ± 60
	Ejection fraction (%)	−8 ± 14	+3.7 ± 16

Shiota et al. [103]	End-diastolic volume (mL)	−43 ± 65	—
	End-systolic volume (mL)	−37 ± 67	—
	Ejection fraction (%)	1 ± 4%	—

Cameli et al. [104]	End-diastolic volume (mL)	−6 ± 11	—
	End-systolic volume (mL)	−4 ± 9	—
	Ejection fraction (%)	2 ± 5%	—

Chan et al. [33]	End-diastolic volume (mL)	−10.4 ± 26.4	—
	End-systolic volume (mL)	−0.9 ± 18.8	—

Sohns et al. [50]	End-diastolic volume (mL)	2.9 ± 12	—
	End-systolic volume (mL)	2.8 ± 7	—
	Ejection fraction (%)	−1 ± 5	—

Sugeng et al. [36]	End-diastolic volume (mL)	−4	—
	End-systolic volume (mL)	−1	—
	Ejection fraction (%)	2	—

Nikitin et al. [105]	End-diastolic volume (mL)	7 ± 28	—
	End-systolic volume (mL)	3 ± 22	—
	Ejection fraction (%)	−1 ± 10	—

Nikitin et al. [105]	End-diastolic volume (mL)	−14 ± 17	−23 ± 29
	End-systolic volume (mL)	−6.5 ± 16	−15 ± 24
	Ejection fraction (%)	−1 ± 6	1 ± 9

Gutiérrez-Chico et al. [106]	End-diastolic volume (mL)	−3 ± 1	—
	End-systolic volume (mL)	2 ± 7	—
	Ejection fraction (%)	0 ± 6	—

Van der Bosch et al. [75]	End-diastolic volume (mL)	−3 ± 12	—
	End-systolic volume (mL)	−12 ± 31	—
	Ejection fraction (%)	−1 ± 7	—

Pouleur et al. [32]	End-diastolic volume (mL)	−20 ± 31	—
	End-systolic volume (mL)	−12 ± 31	—
	Ejection fraction (%)	1 ± 11	—

Qi et al. [107]	End-diastolic volume (mL)	−22 ± 23	—
	End-systolic volume (mL)	−15 ± 20	—
	Ejection fraction (%)	5 ± 10	—

Bicudo et al. [108]	End-diastolic volume (mL)	−4	—
	End-systolic volume (mL)	0.3	—
	Ejection fraction (%)	−2	—

Shimada and Shiota [22]	End-diastolic volume (mL)	−9.9	—
	End-systolic volume (mL)	−4.7	—
	Ejection fraction (%)	−0.13	—

2DE: two-dimensional echocardiography; 3DE: three-dimensional echocardiography; CMR: cardiac magnetic resonance; SD: standard deviation.

**Table 3:** Differences between left ventricular mass calculation by three-dimensional echocardiography and conventional two-dimensional echocardiography in comparison with cardiac magnetic resonance.

Author	Mean difference ± SD from CMR (g)	
	3DE	2DE
Mor-Avi et al. [63]	−4 ± 17	−39 ± 29
Caiani et al. [62]	−2.1 ± 11.5	−34.9 ± 24.8
Jenkins et al. [72]	0 ± 38	16 ± 57
Jian et al. [65]	−9 ± 33	−15 ± 47
Oe et al. [71]	−14.1 ± 29.1	−10.7 ± 83.7
van den Bosch et al. [70]	2 ± 20	—
Bicudo et al. [108]	−6	—
Takeuchi et al. [73]	−2	—
Pouleur et al. [32]	1 ± 3	—

Abbreviations as in Table 2.

**Table 4:** Published reference values for three-dimensional echocardiography derived left ventricular geometry and function parameters.

	Aune et al. 2010 [42]	Fukuda et al. 2012 [40]	Chahal et al. 2012 [44]	Muraru et al. 2013 [41]
Number of subjects	166	410	978	226
Women (%)	52	38	36	55
Ethnicity	Scandinavian	Japanese	51% European white 49% Asian Indian	Italian
Age range (years)	29–80	20–69	35–75	18–75
Setting	Single center	Multicenter (23 centers in Japan)	Clinic rosters of 58 general practitioners	Single center
Echo system vendor(s) and machine model(s)	Philips, iE33	Philips: iE33 and Sonos 7500 and GE: Vivid 7 and E9	Philips iE33	GE Vivid E9
Weight (kg)				
Men	83	66	—	76
Women	69	50	—	61
Body surface area (m^2^)				
Men	2.05	1.8	White: 2.0; Indian: 1.9	1.93
Women	1.78	1.5	White: 1.8; Indian: 1.7	1.66
EDVi (mL/m^2^)				
Men	66 (ULN = 86)	50 (ULN = 64)	White: 49 (ULN = 67) Indian: 41 (ULN = 59)	63 (ULN = 85)
Women	58 (ULN = 74)	46 (ULN = 64)	White: 42 (ULN = 58) Indian: 39 (ULN = 55)	56 (ULN = 72)
ESVi (mL/m^2^)				
Men	29 (ULN = 41)	19 (ULN = 29)	White: 19 (ULN = 29) Indian: 16 (ULN = 26)	24 (ULN = 34)
Women	23 (ULN = 33)	17 (ULN = 25)	White: 16 (ULN = 24) Indian: 15 (ULN = 23)	20 (ULN = 28)
SVi (mL/m^2^)				
Men	—	—	—	39 (LLN = 25)
Women	—	—	—	36 (LLN = 24)
Ejection fraction (%)				
Men	57 (LLN = 49)	61 (LLN = 53)	White: 61 (LLN = 51) Indian: 62 (LLN = 50)	62 (LLN = 54)
Women	61 (LLN = 49)	63 (LLN = 55)	White: 63 (LLN = 53) Indian: 63 (LLN = 53)	65 (LLN = 57)
Mass index (g/m^2^)				
Men	—	64 (ULN = 88)	—	77 (ULN = 57)
Women	—	56 (ULN =78)	—	74 (ULN = 58)
Mass/EDV (g/mL)	—		—	
Men	—	1.3 (ULN = 1.9)	—	1.24 (ULN = 1.60)
Women	—	1.2 (ULN = 1.8)	—	1.30 (ULN = 1.66)

EDVi: end-diastolic volume index; ESVi: end-systolic volume index; LLN: lower limit of normal; SVi: stroke volume index; ULN: upper limit of normal.

Data are reported as mean value (ULN or LLN as specified). LLN and ULN are defined as mean ± 2 standard deviations.

## Abbreviations

2DE: Two-dimensional echocardiography
3DE: Three-dimensional echocardiography
CMR: Cardiac magnetic resonance
CRT: Cardiac resynchronization therapy
CT: Cardiac computerized tomography
ECG: Electrocardiographic
LV: Left ventricle/ventricular
TTE: Transthoracic echocardiography
TOE: Transoesophageal echocardiography.
